# *Kdr* genotyping in *Aedes aegypti* from Brazil on a nation-wide scale from 2017 to 2018

**DOI:** 10.1038/s41598-020-70029-7

**Published:** 2020-08-06

**Authors:** Monique Melo Costa, Kauara Brito Campos, Luiz Paulo Brito, Emmanuel Roux, Cynara Melo Rodovalho, Diogo Fernandes Bellinato, José Bento Pereira Lima, Ademir Jesus Martins

**Affiliations:** 1 Laboratório de Fisiologia e Controle de Artrópodes Vetores, Instituto Oswaldo Cruz, FIOCRUZ, Rio de Janeiro, RJ Brazil; 2 Laboratório de Parasitologia Médica e Biologia de Vetores, Faculdade de Medicina, Universidade de Brasília, Brasília, DF Brazil; 3 Coordenação Geral de Vigilância de Aboviroses, Secretaria de Vigilância em Saúde, Ministério da Saúde, Brasília, DF Brazil; 4 ESPACE-DEV, IRD, Université de Montpellier, Université de La Réunion, Université de la Guyane and Université des Antilles, Montpellier, France; 5 Laboratório Misto Internacional “Sentinela”, FIOCRUZ, UnB, IRD, Rio de Janeiro, Brazil; 6 Instituto Nacional de Ciência e Tecnologia em Entomologia Molecular (INCT-EM), Universidade federal do Rio de Janeiro, Rio de Janeiro, RJ Brazil

**Keywords:** Genetic markers, Entomology, Viral infection

## Abstract

Insecticide resistance is currently a threat to the control of *Aedes agypti*, the main vector of arboviruses in urban centers. Mutations in the voltage gated sodium channel (*Na*_*V*_), known as *kdr* (knockdown resistance), constitute an important selection mechanism for resistance against pyrethroids. In the present study, we investigated the *kdr* distribution for the Val1016Ile and Phe1534Cys alterations in *Ae. aegypti* from 123 Brazilian municipalities, based on SNP genotyping assays in over 5,500 mosquitoes. The alleles *Na*_*V*_*S* (1016Val^+^ + 1534Phe^+^), *Na*_*V*_*R1* (1016Val^+^ + 1534Cys^*kdr*^) and *Na*_*V*_*R2* (1016Ile^*kdr*^ + 1534Cys^*kdr*^) were consistently observed, whereas *kdr* alleles have rapidly spread and increased in frequency. *Na*_*V*_*S* was the less frequent allele, mostly found in Northeastern populations. The highest allelic frequencies were observed for *Na*_*V*_*R1*, especially in the North, which was fixed in one Amazonian population. The double *kdr Na*_*V*_*R2* was more prevalent in the Central-west and South-eastern populations. We introduce the ‘*kdr* index’, which revealed significant spatial patterns highlighting two to three distinct Brazilian regions. The 410L *kdr* mutation was additionally evaluated in 25 localities, evidencing that it generally occurs in the *Na*_*V*_*R2* allele. This nationwide screening of a genetic mechanism for insecticide resistance is an important indication on how pyrethroid resistance in *Ae. aegypti* is evolving in Brazil.

## Introduction

The number of dengue cases in Brazil totaled over 1.9 million records between 2016 and 2018. During the same period, the recent additional chikungunya and Zika arbovirus epidemics were responsible for around 540 and 240 thousand cases, respectively, according to Ministry of Health official bulletins^1^. Failure in the control of *Aedes aegypti*, so far considered the primary vector of the aforementioned arboviruses in Brazil, is considered the main reason for the increase in these records. Despite a series of studies evidencing arbovirus transmission by Brazilian *Aedes albopictus* populations, this species probably acts as a secondary urban vector, mostly significant in maintaining arbovirus circulation during inter-epidemic seasons and in rural regions^2,3^. The measures for controlling the density of *Aedes* mosquitoes largely rely on insecticides. However, their intense use has been, increasingly, selecting resistant populations at a global scale^4^. This is also true in Brazil, where an extensive insecticide resistance monitoring system has tracked the susceptibility status of *Ae. aegypti* populations since 1999^5,6^. Organophosphate application has been intensified since the 1980s, substituted by insect growth regulators and pyrethroids two decades later, against larvae and adults, respectively, given the confirmation of resistance to the larvicide temephos^7^. However, pyrethroid resistance was also confirmed a few years later^8^. Currently, the organophosphate malathion is employed by governmental campaigns in the entire country as the only alternative to pyrethoids, although the latter class of compounds is still intensively used in households and by private companies for mosquito control, as well as against other urban insect vectors, such as *Culex*, sandflies, anophelines and triatomines^6,9,10^.

The main cause of resistance to the pyrethroid knockdown effect is an alteration in the molecular target of this chemical, namely the voltage gated sodium channel (Na_V_, also commonly referred to as VGSC), caused by one or a few amino acid substitutions that alter the channel conformation, thus avoiding its interaction with pyrethroid molecules^11^. These substitutions, known as knockdown resistance mutations (*kdr*) have been linked to pyrethroid and DDT resistance in several arthropod species. The *kdr* amino acid position is generally convergent throughout distinct taxa, as the *Na*_*V*_ gene is very conserved and, therefore, few variations are permissive to maintain its neurophysiological role^12^. In several species, including *Anopheles* and *Culex* mosquitoes, the most classical *kdr* is a Leu to Phe substitution in the 1014 position (L1014F)^13–15^. In the *Aedes* genus, two simultaneous substitutions in the codon 1014 would be necessary, which is unlikely to occur^16^. Substitutions in the 1016 and 1534 sites have been consistently detected in the Na_V_ of *Ae. aegypti* populations worldwide. The Phe to Cys substitution in the 1534 site (F1534C) is found in populations from the Americas, Africa and Asia, whilst the wild-type Val is substituted by Ile (V1016I) in the 1016 site in American populations and by Gly (V1016G) in Asian populations^17–20^. Additional alterations have also been reported, such as the Pro to Ser substitution in the 989 (P989S) and Val to Leu in the 410 position (V410L), respectively, in Asian and Latin American populations^4,17,21,22^.

Since the beginning of the 2000s, *kdr* mutations have been detected in *Ae. aegypti* populations from Brazil, displaying geographical expansion and increasing frequency trends. A haplotype with *kdr* mutation F1534C (*Na*_*V*_*R1* allele) was present in all but one population among natural populations evaluated from more than 60 localities^6, 23^. An additional haplotype containing both V1016I and F1534C was absent or detected at very low frequencies in the Brazilian Northeast^9,23,24^. Although this double *kdr* allele (*Na*_*V*_*R2*) presented a high fitness cost in a pesticide-free environment^25^, its frequency has increased in natural populations and dispersed to localities where it was recently considered absent^26^. *kdr* mutations were monitored in *Ae. aegypti* populations from the state of São Paulo, since they were present at low frequencies, and, although *Na*_*V*_*R2* later appeared, it increased faster than *Na*_*V*_*R1*^9^. This can be explained by the fact that *Na*_*V*_*R2* confers a higher level of resistance to pyrethroids^27^.

Additional variations have been reported in the *Na*_*V*_ gene of *Ae. aegypti* populations from Brazil, including the Ile to Met and Val to Leu substitutions in the 1011 (I1011M) and 410 (V410L) positions, respectively. The former is related to a duplication in the *Na*_*V*_ gene, always detected in heterozygote individuals^28^, but with no relevance in pyrethroid resistance^27^. On the other hand, V410L was found alone or in combination with F1534C, conferring high levels of resistance^29^, although it is usually found in combination with V1016I and F1534C in Mexican populations^21^. The present study reports the *kdr* frequencies in Brazilian *Ae. aegypti* populations from 123 localities, collected between 2017 and 2018. In order to rank the likely levels of pyrethroid resistance among the evaluated populations, at least in relation to the target site mechanism, an original index called the *kdr index* was developed. A spatial analysis confirmed the non-randomness of the spatial distribution of the index values and, thus, the existence of clearly identifiable Brazilian regions regarding with pyrethroid resistance levels.

## Results

### *Kdr* genotyping

**Figure 1:**
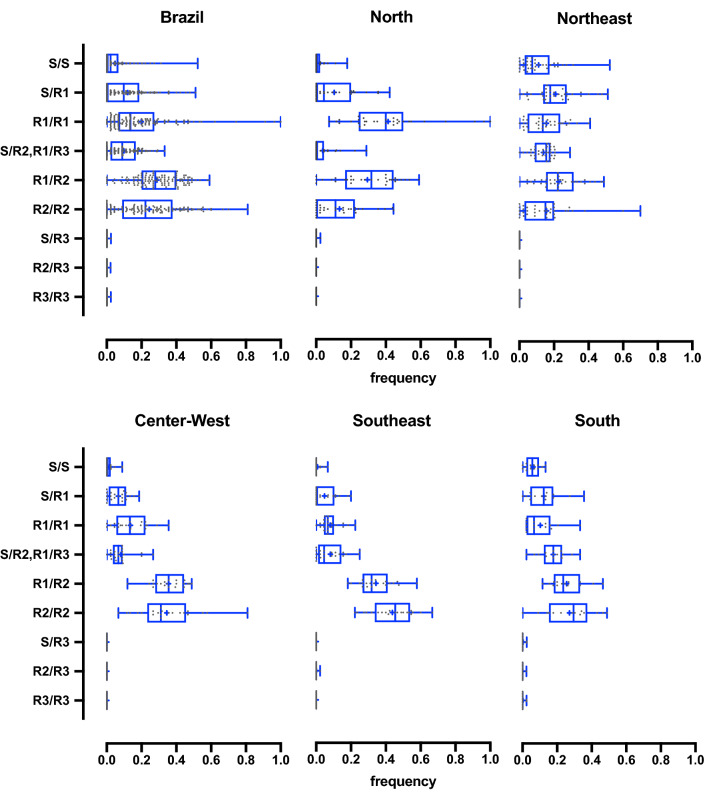
Frequency of *kdr* genotypes in *Aedes aegypti* from Brazil considering the V1016I and F1534C variations in the voltage gated sodium channel. Populations were distributed according to the Brazilian five macro-geographical regions. Dots represent the genotypic frequency of each population, while the box-plots exhibit the median, quartiles, minimum and maximum values. Genotypes: 1016 + 1534.

The *kdr* genotypes for 5,341 individuals, distributed in 123 localities, for both the 1016 and 1534 Na_V_ sites were obtained. All evaluated population displayed at least one *kdr* allele. Six genotypes were substantially observed, all including the *S*, *R1* and *R2* (wild-type, *kdr* in 1534 only and *kdr* in 1016 + 1534, respectively) haplotypes. The other three genotypes composed by the *kdr R3* haplotype (*kdr* in 1016 site only) accounted for 0.1% of the total samples (Fig. 1 and Table 1). Therefore, the *R3* haplotype was not considered in further analyses.

**Table 1:** Genotypes of 123 *Aedes aegypti* populations from Brazil, considering the V1016I and F1534C SNPs in the *Na*_*V*_ gene.

PCR results	Genotypes	Number (frequency)^a^	Median	Min^b^	Max^c^
VV + FF	SS	255 (0.048)	0.022	0	0.523
VV + FC	SR1	652 (0.122)	0.098	0	0.511
VV + CC	R1R1	1,054 (0.197)	0.136	0	1.000
VI + FC	SR2, R1R3	524 (0.098)	0.089	0	0.333
VI + CC	R1R2	1,548 (0.290)	0.279	0	0.591
II + CC	R2R2	1,301 (0.244)	0.222	0	0.810
VI + FF	SR3	3 (0.0006)	0.000	0	0.024
II + FC	R2R3	2 (0.0004)	0.000	0	0.022
II + FF	R3R3	2 (0.0004)	0.000	0	0.023

^a^Number of samples with their respective genotypes, ^b^Minimum and ^c^Maximum observed frequencies.

**Figure 2:**
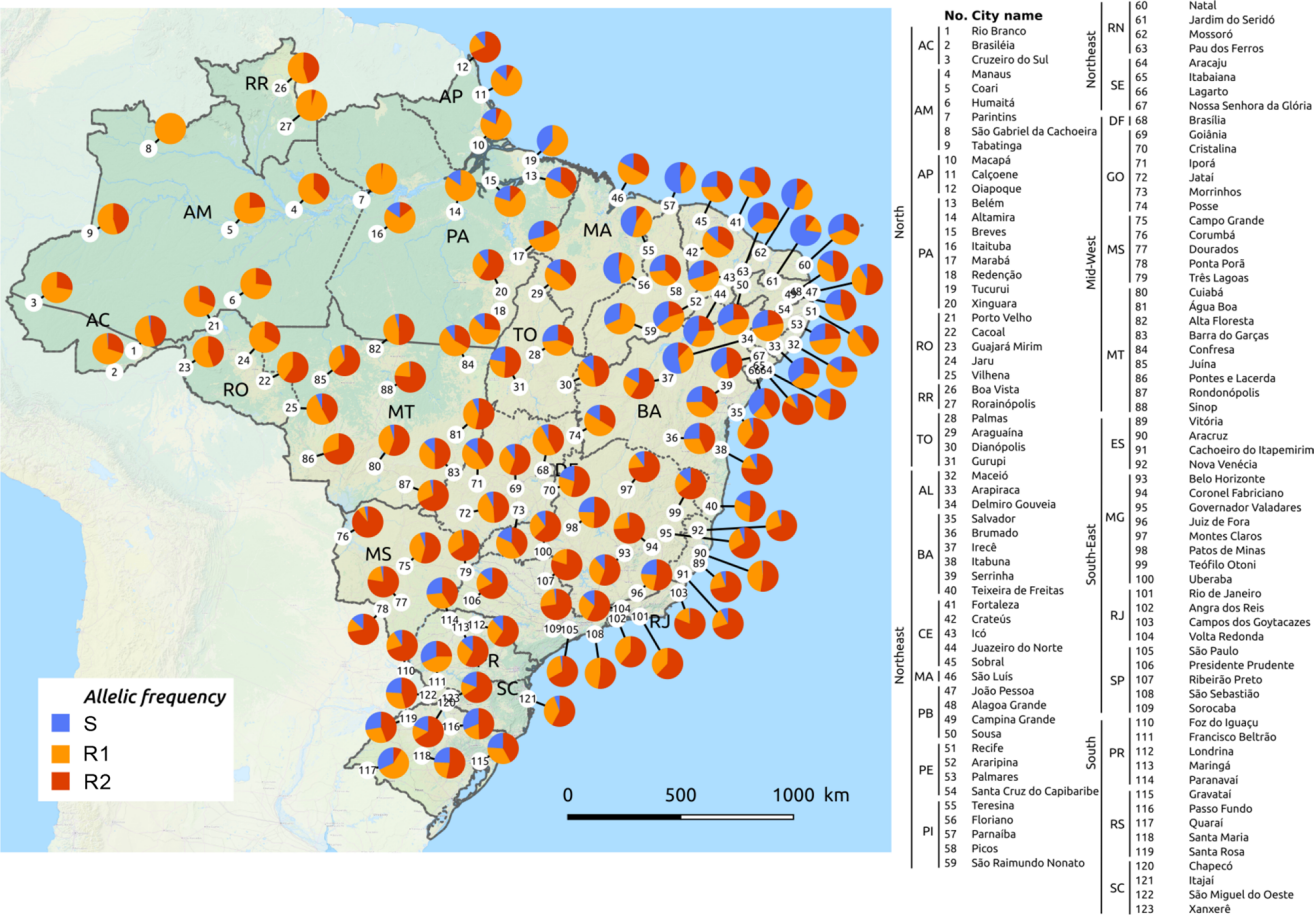
Distribution of the *kdr* allelic frequencies in *Aedes aegypti* populations from Brazil considering the V1016I and F1534C variations in the voltage gated sodium channel. Localities are represented by numbers in the legend, exhibited on the map according to their respective geographical coordinates, grouped within the five Brazilian macro-regions. The background map is the “OSM TF Landscape” product (Maps Thunderforest, Data OpenStreetMap contributors, under license CC-BY-SA 2.0). Map was generated with the free and open source software (GNU General Public License) QGIS version 3.12.3 (developd by the Open Source Geospatial Foundation Project, http://qgis.org).

**Figure 3:**
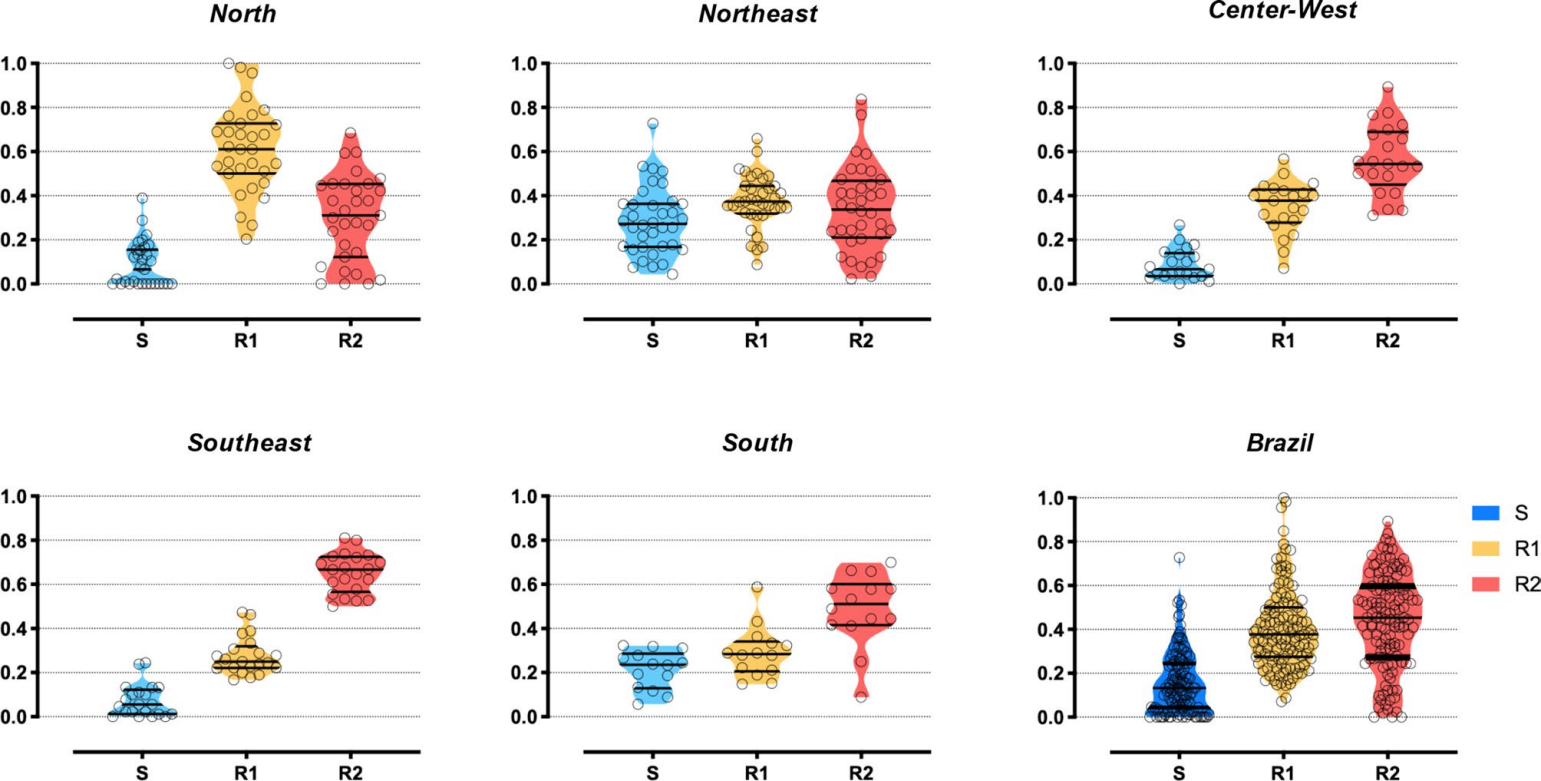
Frequency of the *kdr* alleles in *Aedes aegypti* from Brazil considering the V1016I and F1534C variations in the voltage gated sodium channel. Plots represent the *Na*_*V*_ allelic frequency distribution of each population in the five geographic Brazilian macro-regions and in the country as a whole. The alleles are *Na*_*V*_*S* (V1016 + F1534), *Na*_*V*_*R1* (V1016 + 1534C) and *Na*_*V*_*R2* (1016I + 1534C). Circles represent the allelic frequency of each population and bars indicate the median and quartile distribution of the respective allele.

The most frequent *Ae. aegypti* genotypes was R1R2, followed by the double *kdr* homozygote R2R2, with medians of 29.0% and 24.4%, respectively (Table 1). The genotype frequencies per population in their respective geographic region are displayed in Fig. 1. Concerning allelic frequencies, medians were 13.3%, 37.8% and 45.3%, respectively for *Na*_*V*_*S*, *Na*_*V*_*R1* and *Na*_*V*_*R2*, evidencing the high prevalence of *kdr* alleles (83.1%) in Brazil. The wild-type allele *Na*_*V*_*S* was absent from 17 of the 123 evaluated populations, while *kdr Na*_*V*_*R1* was present in all localities, ranging from 7.1% (Corumba/MS) to 100% (São Gabriel da Cachoeira/AM) and the *kdr Na*_*V*_*R2* ranged from absent (Tucurui/PA, Altamira/PA and São Gabriel da Cachoeira/AM) to 89.3% (Corumba/MS) (Fig. 2). The *kdr* allelic frequencies were regionalized in the country, as a reflection of their genotypic distribution, as follows: *Na*_*V*_*R2* predominated in the Central-West, Southeast and South regions, while *Na*_*V*_*R1* was the most frequent allele in the North (Fig. 3). The detailed genotypic and allelic frequencies for each locality are available in the Supplementary Table S1.

### *Kdr* index and spatial analyses

**Figure 4:**
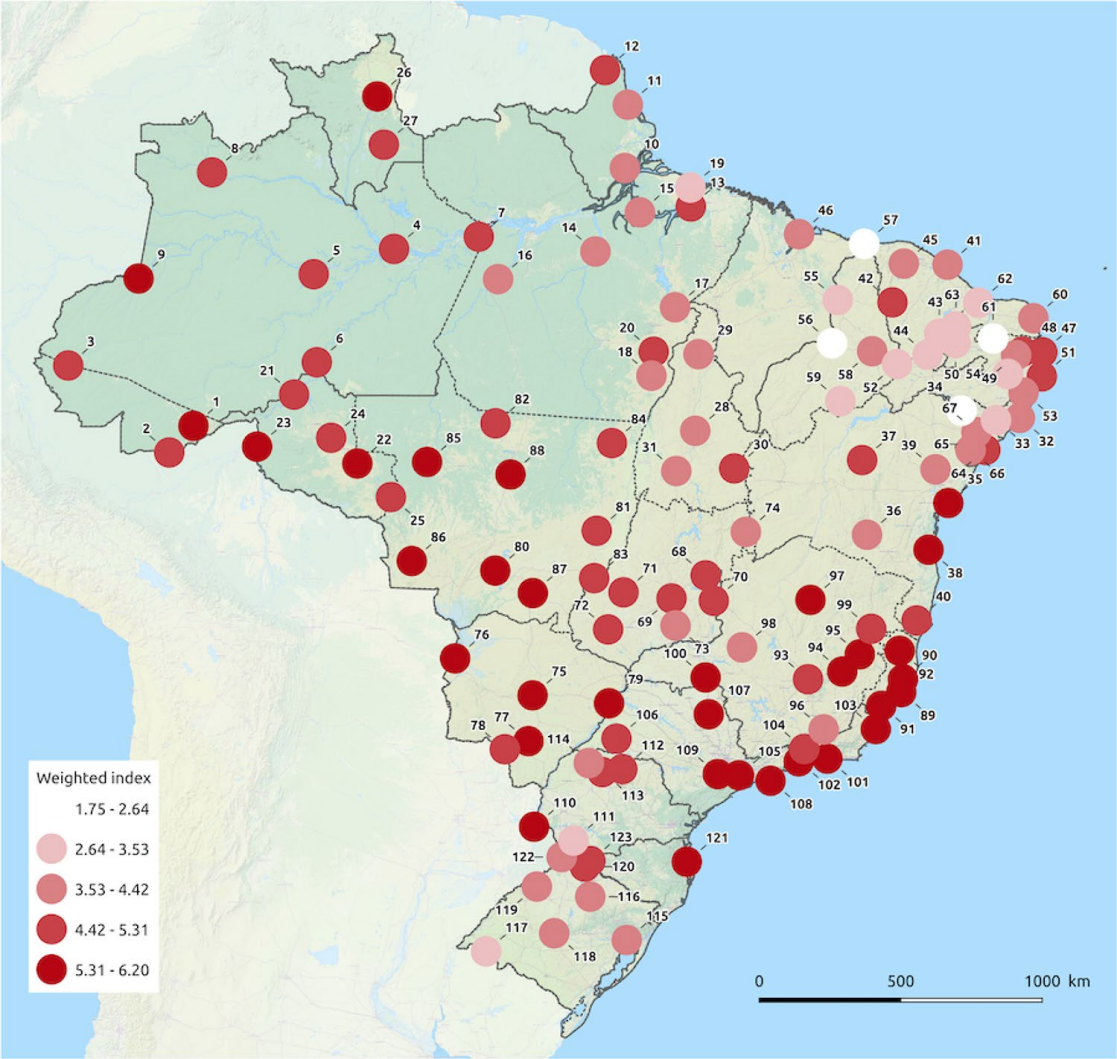
*kdr index* distribution in *Aedes aegypti* from Brazil. This index represents the *kdr* genotypic frequency weighted by the deltamethrin *knockdown time* (*Kd*T RR_95_) for the respective genotype, as determined elsewhere^27^. Locality identification is similar to that exhibited in Fig. 2. The background map is the “OSM TF Landscape” product (Maps Thunderforest, Data OpenStreetMap contributors, under license CC-BY-SA 2.0). Map was generated with the free and open source software (GNU General Public License) QGIS version 3.12.3 (developd by the Open Source Geospatial Foundation Project, http://qgis.org).

The ‘*kdr index*’ ranged from 1.75 (Jardim do Seridó, RN) to 6.21 (Corumbá, MS) (Fig. 4). The localities presenting the lowest *kdr* index (first quartile: 1.75 to 3.96) were predominantly located in the Northeast (61.3%), South (19.4%) and North (16.1%) regions. Those beyond the last quartile (5.36–6.21) were distributed in the Southeast (48.4%), Center-West (25.8%), North (12.9%), Northeast (9.7%) and South (6.5%) regions (Supplementary Table S1).

**Figure 5:**
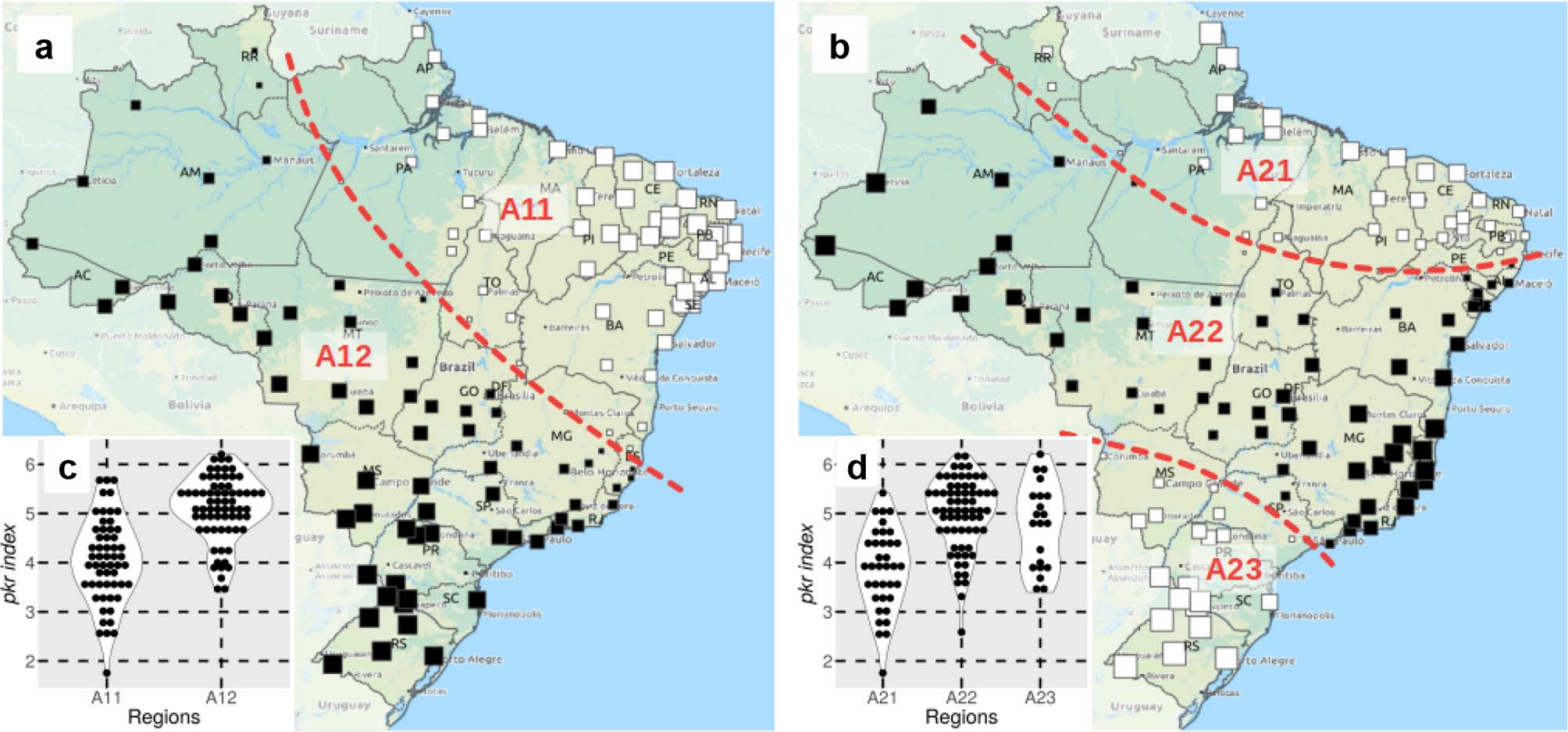
Results of the *kdr index* value distribution spatial analysis. (**a**) and (**b**) indicate the spatial patterns and the corresponding regions identified from the *kdr index* value spatial distributions in Brazil for *Aedes aegypti*. These patterns correspond to the most explanatory eigenvectors of a principal coordinates analysis of a given neighbor matrix (see Supplementary Text S1). White and black squares consist of negative and positive eigenvector component values, respectively, and square size is proportional to the absolute value of the vector components. These patterns were selected because they present a significant Moran’s I spatial autocorrelation and explain 51.6% (29.6% and 22.0% for A and B, respectively) of the total variance of the observed *kdr index* values, by their inclusion as explanatory variables in a multiple linear regression model. (**c**) and (**d**) display the *kdr index* value distributions as a function of the identified regions. The background map is the “OSM TF Landscape” product (Maps Thunderforest, Data OpenStreetMap contributors, under license CC-BY-SA 2.0). Map was generated with the free and open source software (GNU General Public License) QGIS version 3.12.3 (developd by the Open Source Geospatial Foundation Project, http://qgis.org).

The spatial distribution *kdr* index value analysis concerning the geographic coordinates of their respective localities revealed statistically significant spatial patterns, i.e. the existence of clearly identifiable Brazilian regions with different predicted *Ae. aegypti* resistance status. A total of 55 candidate models, related to different (a priori) inter-city “links” structures, were generated (see Supplementary Text S1 for methodological details and intermediary results). Figure 5 displays the most significant model, first evidencing a large scale spatial differentiation in the Northeast–Southwest direction, separating Northeast (and to a lesser extent, North) localities from the rest of the country (areas A11 and A12, respectively, represented in Fig. 5a and, second, a three-mode differentiation in the same direction, separating: (i) North and Northeast localities; (ii) a strip of cities oriented Southeast–Northwest and (iii) South localities (areas A21, A22 and A23, respectively, in Fig. 5b. The *kdr index* value distributions as a function of the aforementioned areas are displayed in Fig. 5c,d.

### Including the V410L SNP

**Figure 6:**
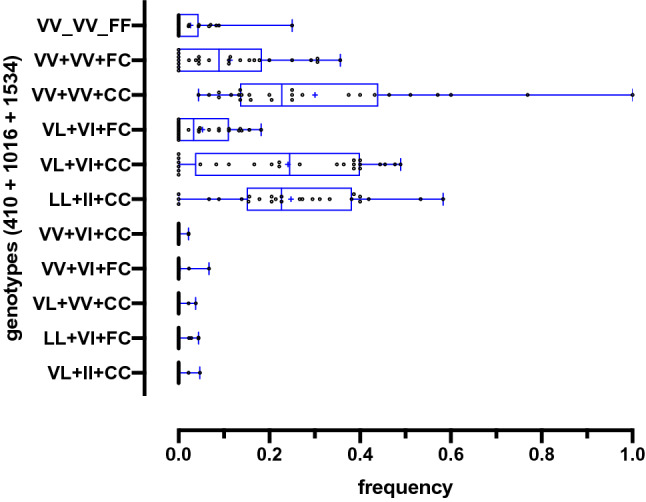
Frequency of *kdr* genotypes in *Aedes aegypti* from Brazil, considering the 410, 1016 and 1534 sites in the voltage gated sodium channel. Dots represent the allelic frequency of each population and bars indicate the median and quartile distribution of the respective allele.

In addition to V1016I and F1534C, the V410L SNP was also evaluated in 25 populations, mostly in the state capitals, totaling 977 genotyped samples for these three sites, which together rendered 11 genotypes, amongst which five were poorly represented (Table 2, Fig. 6). The three most prevalent genotypes were VV + VV + CC (27.8%), VL + VI + CC (26.8%) and LL + II + CC (23.9%) (in order, 410 + 1016 + 1534), composed by the *kdr* haplotypes *VVC* (*kdr* in the 1534 site only) and *LIC* (*kdr* in 410, 1016 and 1534 sites). Next are the genotypes formed with the wild-type haplotype (*VVF*): VV + VV + FC (11.1%), VL + VI + FC (6.0%) and VV + VV + FF (2.7%). The remaining five genotypes occurred in frequencies below 1%, which would be formed by alleles such as *VIC* (*kdr* in 1016 and 1534), *LVC* (*kdr* in 410 and 1534), *LVF* (*kdr* in 410 only) and *VIF* (*kdr* in 1016 only). Considering only samples with the six best-represented genotypes, the haplotypic frequencies were calculated as 40.0% (*VVC*), 38.2% (*LIC*) and 10.4% (*VVF*), indicating that 410L was associated with 1016I and 1534C (*Na*_*V*_*R2* allele) and that 1534C also occurs alone (*Na*_*V*_*R1* allele). Nevertheless, we cannot exclude the possibility that the underrepresented genotypes could be composed by additional haplotypes circulating under low frequencies. The complete dataset displaying the numbers and frequencies for each SNP in all populations is presented in Supplementary Table S2.

**Table 2:** Genotypes of 25 *Aedes aegypti* populations from Brazil, considering the V410L, V1016I and F1534C SNPs in the *Na*_*V*_ gene.

PCR results (410 + 1016 + 1534)	Genotypes	Number (frequency)^a^	Median	Min^b^	Max^c^
VV + VV + FF	VVF/VVF (SS)	26 (0.027)	0	0	0.250
VV + VV + FC	VVF/VVC (SR1)	108 (0.111)	0.089	0	0.356
VV + VV + CC	VVC/VVC (R1R1)	272 (0.278)	0.227	0.044	1.000
VL + VI + FC	VVF/LIC (SR2), VVC/LIF (R1R3)	59 (0.060)	0.034	0	0.182
VL + VI + CC	VVC/LIC (R1R2)	262 (0.268)	0.244	0	0.489
LL + II + CC	LIC/LIC (R2R2)	233 (0.239)	0.227	0	0.583
VV + VI + CC	VVC/VIC	2 (0.002)	0	0	0.022
VV + VI + FC	VVF/VIC, VVC/VIF	4 (0.004)	0	0	0.067
VL + VV + CC	VVC/LVC	2 (0.002)	0	0	0.038
LL + VI + FC	LVF/LIC, LVC/LIF	6 (0.006)	0	0	0.044
VL + II + CC	VIC/LIC	3 (0.003)	0	0	0.047

^a^Number of samples with their respective genotypes, ^b^Minimum and ^c^Maximum observed frequencies.

## Discussion

The molecular nation-wide monitoring of the main pyrethroid resistance marker in 123 *Ae. aegypti* populations from Brazil, a country comprising 8.5 million km^2^, where the 26 federal states are infested with this vector, is presented herein. To the best of our knowledge, this is the largest *kdr* survey performed using samples collected in the time span of one year. The regionalized profile of allelic frequency distribution indicates that this sample size is enough to represent the whole country.

The frequency of *kdr* alleles was very high in Brazil (median of 83.1%), representing an increase of 27.8% since a previous survey was performed, evaluating 29 localities where mosquitoes were collected between 2009 and 2012^14^. The *kdr Na*_*V*_*R1* allele (1016Val + 1534Cys^*kdr*^) was present in all Brazilian populations evaluated here, and fixed in one of them. This was probably the first *kdr* allele to spread in Latin America^20^ and is present in at least two haplotypes of independent phylogenetic origins^17^. *kdr Na*_*V*_*R2* (1016Ile^*kdr*^ + 1534Cys^*kdr*^) confers a higher level of resistance to pyrethroids compared to *Na*_*V*_*R1*^17^, which may explain why *Na*_*V*_*R2* frequency is rapidly increasing compared to the wild-type *Na*_*V*_*S* and *kdr Na*_*V*_*R1* alleles, as observed in *Ae. aegypti* populations from São Paulo^9^. It is interesting to note that, out of the 17 populations where the wild-type *Na*_*V*_*S* was absent, 12 were from the Amazonian region. In many of these localities, chemical control is not only exerted to combat *Ae. aegypti*, but also targeting other important vectors, such *Anopheles* mosquitoes, triatomines and phlebotomines, against which pyrethroids are the main choice inside houses and in the peri-domicile area. Therefore, the scenario observed herein may be explained by this constant selection pressure.

We also presented the application of a ‘*kdr index*’ which considers the *kdr* genotype frequencies weighed by their respective *knockdown* time resistance ratio (*Kd*T RR_95_). The lowest *kdr* indexes were predominately found in populations from the Northeast, where the *kdr Na*_*V*_*R2* allele was almost absent from the previous survey^24^ and is now present in all states in that region, with frequencies ranging from 10.2% (Jardim do Seridó, RN) to 83.8% (Itabaiana, SE). In fact, in a previous study assessing resistance to the pyrethroid deltamethrin amongst the 13 populations with pyrethroid resistance ratios (RR_95_) below 10, reported that seven were located in the Northeast region. In addition, only two populations in the Northeast were amongst the 24 highly resistant populations (RR_95_ > 10)^6^.

The study of the spatial distribution of resistance markers can aid in identifying underlying resistance emergence processes in surrounding areas. In fact, these determinants can exhibit significant “spatial patterns”, due to their strong dependency to space, climate, land cover and land use, active and passive mosquito mobility between connected cities and regional strategies for vector control, among others. These patterns may display spatial variations at different scales. As a consequence, a spatial distribution of the resistance level results of the mixture of these different multi-scale spatial patterns is observed. The decomposition of the observed level of resistance into various independent and significant spatial patterns can, consequently, not only exhibit spatial clusters of cities that present significantly high (or low) resistance levels, but also highlight spatial patterns typical of phenomena that may, therefore, be considered as potential determinants. In the present study, we tested a series of analyses considering the spatialized *kdr* index values, revealing two to three well defined clustered populations in the country. This evidenced that the genetic trend of higher or lower pyrethroid resistant (the *kdr index*) is not random in Brazil. The populations placed in the A12 or A22 areas, in the models with two or three clusters, respectively, are more likely to be resistant to pyrethroids than the rest of the country, considering the target site mechanism. On the other hand, specific conditions may lead to diverse insecticide resistance patterns, including distinct *kdr* allelic frequencies, as observed in *Ae. aegypti* populations from five neighborhoods in Merida, Mexico^30^. New studies including spatial patterns details will further knowledge on the dynamics of insecticide resistance distribution and expansion in natural populations.

The most frequent genotype in *Ae. aegypti* from Brazil was R1R2, which was heterozygous for the alleles *Na*_*V*_*R1* and *Na*_*V*_*R2*, in 28.9% of all evaluated samples. The second most frequent, at 24.4%, was the double *kdr* R2R2, homozygous for *Na*_*V*_*R2*, followed by R1R1 in 19.9%, homozygous for *Na*_*V*_*R1*. The sum of all “resistant genotypes”^27^ indicated that 73.2% of all *Ae. aegypti* from Brazil evaluated herein would be resistant to pyrethroid, at least considering the target-site resistance mechanism. It is worth noting, however, that metabolic resistance might also contribute, alone or in synergism with *kdr*, to confer pyrethroid resistance in the natural populations of this country^6^. Although the organophosphate malathion began replacing pyrethroids in official campaigns oriented by the Brazilian MoH in 2009^31^, the selection pressure of pyrethroids is still heavily present in *Ae. aegypti* due to household aerosol insecticide products^9,32,33^. Experiments conducted in Merida, Mexico, indicate that 87% of households regularly used pyrethroid-based products against mosquitoes^33^ and that *Ae. aegypti* field populations were resistant to commonly household used products. In addition, an increase in *kdr* 1016Ile was associated with the employment of pyrethroid surface sprays in houses^34^.

*kdr* mutations, or at least the *kdr Na*_*V*_*R2* allele, have a high fitness cost and tend to decrease in insecticide-free environments^25^. In a very practical example, the first *Ae. aegypti* lineage infected with *Wolbachia* released in Rio de Janeiro was actually a result of backcrosses between an original infected Australian lineage and a natural Brazilian population, therefore resulting in a *Wolbachia*-infected lineage resistant to pyrethroids. However, during several generations in the laboratory awaiting governmental licenses for release, *kdr* frequencies decreased and the lineage became susceptible^32^. As residents were spraying pyrethroid-based aerosols in the study area, *Wolbachia* dispersion did not occur as expected and the first release failed. Thereafter, a new lineage was again backcrossed with field mosquitoes in order to guarantee similar conditions to the well-adapted field population, which then allowed *Wolbachia* to expand and stabilize in the study area. The *kdr* frequency is now monitored in the laboratory lineage and in the target field population in the World Mosquito Program^32^.

The *kdr* SNP V410L was first detected in *Ae. aegypti* samples from Northern and Southeastern Brazil^29^, also observed in samples from Mexico since 2002^21^, where it is currently highly disseminated^35^. The V410L SNP was detected in several of our evaluated samples, with strong association of the mutant variation (410Leu) with 1016Ile (and, therefore, with 1534Cys), i.e. the triple *kdr* allele. In a few samples however, 410L was dissociated from these other *kdr* mutations, as evidenced by individuals genotyped as LL + VI + FC, VL + VV + CC and VL + II + CC (all in the order 410 + 1016 + 1534), which account for only 1.1% of the total. Similar results were also observed in samples from Mexico, where mutations in 410 and 1016 were always associated, or, otherwise, dissociated in very low frequencies^35^. In order to save consumables in a broad *kdr* surveillance in *Ae. aegypti* in Latin American countries, where resources are limited, we recommend that the 1016 site may be genotyped first, and for samples genotyped as 1016 V/V or V/I, a second reaction for the 1534 SNP should be performed. On the other hand, when the samples are detected as 1016 I/I, their genotype are likely to be R2R2, i.e. homozygous for the three *kdr* mutations. Thus, it would not be necessary to genotype the 410 site at all. For academic purposes however, it would be interesting to track possible fluctuations in the frequency of these rare genotypes over time.

New methods for controlling *Ae. aegypti* or at least making the species “immune” to arbovirus infections are being tested or have already been implemented as actual control methods^36^. The use of insecticides however may be maintained, due to their potential of rapidly decreasing the density of a targeted population, unless resistance to the applied compound is present. New products with active neonicotinoid-class ingredients have been recently approved by WHO PQT-VC, namely clothianidin, as an indoor residual spray (IRS), and imidacloprid, as an ultra-low volume (ULV) compound^37^. Although these neonicotinoids are slower-acting than pyrethroids and organophosphates, resistant populations to these insecticides seem to not present cross-resistance^38^, indicating them as promising alternative adulticides. In Brazil, the most recent nation-wide *Ae. aegypti* IR monitoring detected several populations resistant to malathion^39^. The Brazilian MoH is currently planning the implementation of an alternative compound comprising a mixture of pyrethroids and clothianidin to be sprayed inside houses, in addition to another compound comprising a mixture of pyrethroids and imidacloprid as an ULV for outdoor areas. On the other hand, a deeper discussion should consider environmental issues regarding neonicotinoids in non-target organisms^40^. In meanwhile, new arbovirus case records in 2019 in Brazil consisted of over 1.5 million dengue cases, 130 thousand chikungunya cases and 10 thousand Zika cases^1^. In Brazil, as well as in several other countries with similar climate and urban landscape conditions, the implementation of new vector control tools is paramount.

## Material and methods

Collections were performed in the context of the national Brazilian insecticide resistance monitoring program for *Ae. aegypti* between 2017 and 2018. Field agents installed eggtraps randomly distributed according to the total number of houses in each municipality, as follows: 100, 150, 200 or 300 traps for < 50, 50–200, 200–500 and > 500 thousand houses, respectively. The installation and collection methodology of the eggtraps is described elsewhere^39^. A presential meeting was organized with field-personnel representatives of each state and a video with step-by-step procedures was launched (https://www.youtube.com/watch?v=2w89kagSOKM) in order to ensure that collections were made as homogeneously as possible. The palettes were sent to the laboratory, where the eggs were stimulated to hatch and larvae were raised up to adults in order to produce an F1 colony of each locality, as previously described^21^. After 4–5 days in the cages, enough time for copulation, a sampling of this F_0_ generation of each population, around 45 insects, preferentially males, were removed and saved for *kdr* genotyping. These mosquitoes were maintained in 80% ethanol solution or cryopreserved prior to DNA extraction. The DNA was extracted from single mosquitoes using the FastDNA Spin (MP Biomedicals) or Nucleo Spin Tissue (Macherey–Nagel) kits, according to their manufacturers' protocol. The eluted DNA was diluted to 10 ng/µL in extra-pure water and cryopreserved until use.

We performed independent genotyping reactions for each *kdr* site based on a qPCR approach using the Custom TaqMan SNP Genotyping Assay (ThermoFisher) (see Table 3 for the primer and probe sequences for each assay). Reactions consisted of 1X TaqMan Genotyping Master Mix (ThermoFisher), 1X of the respective Custom TaqMan SNP Genotyping Assay, 20 ng of DNA and ultra-pure water q.s. 10 µL, run in a QuantStudio 6 Flex (Applied Biosystems), under standard conditions: 45 cycles with a DNA denaturation step (95 °C for 15 s) and primer and probe annealing, followed by DNA polymerization (60 °C for 1 min). The genotypes were obtained by the online software Genotype Analysis Module V3.9 (Applied Biosystems, ThermoFischer cloud platform). The *kdr* sites 1016 (V1016I) and 1534 (F1534C) were evaluated in all populations. For 25 out of the 123 populations, mostly from state capitals, the 410 SNP (V410L) was also assessed.

**Table 3:** Primer and probe sequences for the SNPs V410L, V1016I and F1534C *kdr* in *Aedes aegypti.*

Na_V_ site	Assay ID^a^	Variation	Primers	Probes
410	AN2XA9W	GTA/TTA	For: GTGGCACATGCTCTTCTTCATT	Val: VIC-TCGTTCTACCTTGTAAATT-NFQ
		(Val/Leu)	Rev: GGCGACAATGGCCAAGATC	Leu: FAM-TTCGTTCTACCTTTTAAATT-NFQ
1016	AHS1DL6	GTA/ATA	For: CGTGCTAACCGACAAATTGTTTCC	Val: VIC-CCCGCACAGGTACTTA-FAM
		(Val/Ile)	Rev: GACAAAAGCAAGGCTAAGAAAAGGT	Ile: FAM-CCGCACAGATACTTA-NFQ
1534	AHWSL61	TTC/TGC	For: TCGCGAGACCAACATCTACATG	Phe: VIC-AACGACCCGAAGATGA-NFQ
		(Phe/Cys)	Rev: GATGATGACACCGATGAACAGATTC	Cys: FAM-ACGACCCGACGATGA-NFQ

^a^Identification of the customized TaqMan SNP Genotyping Assay (ThermoFischer).

**Figure 7:**
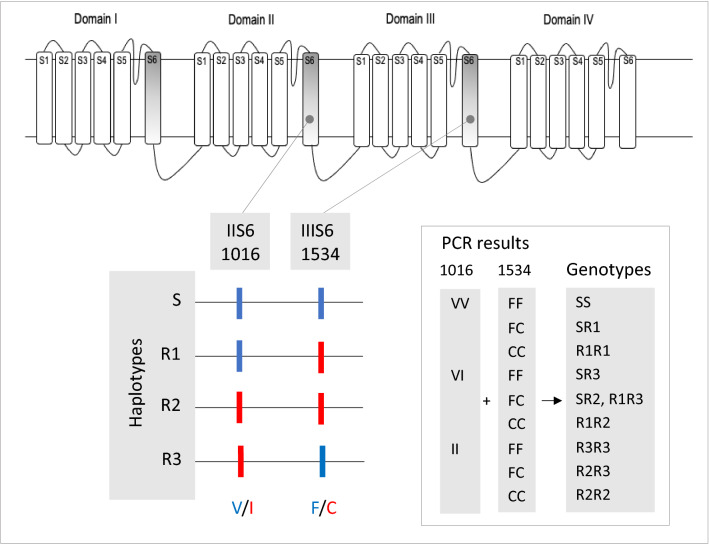
Representation of the *kdr* genotype based on PCR reactions for the 1016 (Val^+^, Ile^*kdr*^) and 1534 (Phe^+^, Cys^*kdr*^) variations in the voltage gated sodium channel of *Aedes aegypti*.

Considering that the evaluated SNPs are linked in the same gene, the genotype of each individual contemplated the results of each site, 1016 (VI, II) and 1534 (FC, CC), resulting in the total combination of nine possible genotypes, composed by four possible haplotypes, *S*, *R1*, *R2* and *R3*^14,22^, as displayed in Fig. 7. For the 25 populations in which the 410 site (VV, VL, LL) was also genotyped, a total of 27 combined genotypes and, consequently, eight haplotypes: *VVF*, *VVC*, *VIC*, *VIF*, *LVF*, *LVC*, *LIC* and *LIF*, were possible.

We ranked the populations in relation to their predicted level of resistance to pyrethroids, based on the *kdr index*, consisting of the sum of *kdr* genotype frequencies weighed with their respective resistance ratios, which were previously obtained in a knockdown time test (*Kd*T RR_95_, Brito et al.^27^. The *Kd*T RR_95_ values for the genotypes were SS (1), SR1 (2.4), SR2 (1.7), R1R1 (4.6), R1R2 (5.4) and R2R2 (6.7). The formula for the *kdr index* of a population is:

$$\begin{aligned} & \left[ {{\rm{f}}\left( {{\rm{SS}}} \right) \times 1} \right] + \left[ {{\rm{f}}\left( {{\rm{SR}}1} \right) \times 2.4} \right] + \left[ {{\rm{f}}\left( {{\rm{SR}}2} \right) \times 1.7} \right] \\ & \quad + \left[ {{\rm{f}}\left( {{\rm{R1R}}1} \right) \times 4.6} \right] + \left[ {{\rm{f}}\left( {{\rm{R1R2}}} \right) \times 5.4} \right] + \left[ {{\rm{f}}\left( {{\rm{R2R}}2} \right) \times 6.7} \right] \end{aligned}$$

In order to test the existence of significant multi-scale spatial patterns (according to Moran’s Index of spatial autocorrelation) regarding the spatial distribution of the *kdr* frequencies, we considered the aforementioned *kdr index* and the geographic coordinates of each locality in a series of spatial statistic tests, applying a principal coordinate analysis of neighbor matrices and Moran’s eigenvector maps^41^, as detailed in Supplementary Text 1.

## Supplementary information

Supplementary Information 1.

Supplementary Information 2.

Supplementary Information 3.

Supplementary Information 4.

## Data Availability

A table with the genotyping data of all populations can be accessed in the supplementary files. Further data and materials are available from the corresponding author upon reasonable request.
