# Supplementary material for: Kdr genotyping in Aedes aegypti from Brazil on a nation-wide scale from 2017 to 2018
Source: Sci Rep. 2020 Aug 6;10:13267. doi: 10.1038/s41598-020-70029-7 (PMC7414026; doi:10.1038/s41598-020-70029-7)

***Kdr* genotyping in *Aedes aegypti* from Brazil on a nation-wide scale from 2017 to 2018**

Monique de Melo Costa, Kauara B. Campos, Luiz Paulo Brito, Emmanuel Roux, Cynara de Melo Rodovalho, Diogo Fernandes Bellinato, José Bento Pereira Lima, Ademir Jesus Martins

Supplementary information – Figure S1 - **Frequency of *kdr* alleles in *Aedes aegypti* from Brazil, considering the variations V1016I and F1534C in the voltage gated sodium channel**. Plots represent the Na_V allelic frequency distribution of each population in the three geographic clusters (please, see Supplementary Text S1). Alleles are *Na_VS* (V1016 + F1534), *Na_VR1* (V1016 + 1534C) and *Na_VR2* (1016I + 1534C). Dots represent the allelic frequency of each population and bars the median and quartiles distribution of the respective allele.

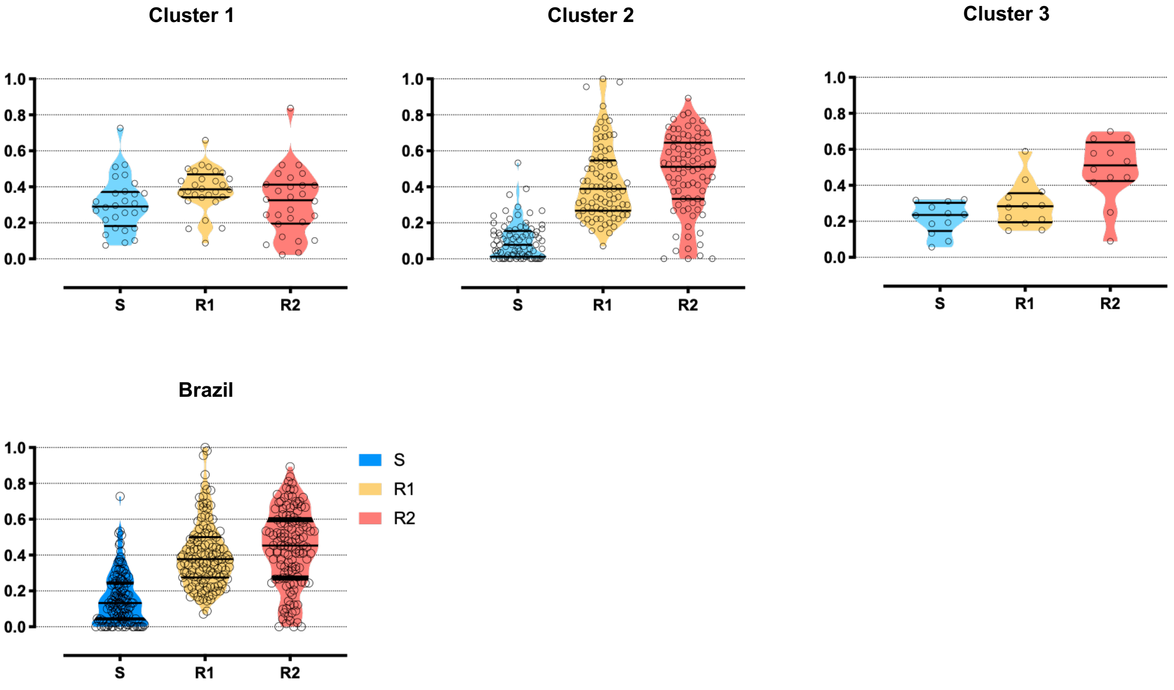

Supplement: Supplementary file 3 — Supplementary Information 3. [file 41598_2020_70029_MOESM3_ESM.pdf]
