# Supplementary material for: Kdr genotyping in Aedes aegypti from Brazil on a nation-wide scale from 2017 to 2018
Source: Sci Rep. 2020 Aug 6;10:13267. doi: 10.1038/s41598-020-70029-7 (PMC7414026; doi:10.1038/s41598-020-70029-7)
Supplement: Supplementary file 4 — Supplementary Information 4. [file 41598_2020_70029_MOESM4_ESM.pdf]

Kdr genotyping in _Aedes aegypti_ from Brazil on a nation-wide scale from 2017 to 2018

Monique de Melo Costa, Kauara B. Campos, Luiz Paulo Brito, Emmanuel Roux, Cynara de Melo Rodovalho, Diogo Fernandes Bellinato, José Bento Pereira Lima, Ademir Jesus Martins

Supplementary Text S1 – Spatial Statistical analyses

Introduction

The spatial distribution of the *kdr index* values partly results of a mixture of the effects of many factors and their interactions at different spatial scales. In fact, climate, land cover and land use, active and passive mosquito mobility between connected cities, regional strategies for vector control, etc. potentially impact selection of resistant *Aedes* populations to insecticides, and exhibit significant spatial auto-correlations due to their strong dependency to space.

In that sense, attempt to decompose the spatialized *kdr index* values in various independent components exhibiting significant spatial auto-correlation – components also referred to as *spatial patterns* hereafter –, can: exhibit regions with significantly high (or low) resistance levels; highlight spatial patterns typical of the resistance determinants. Here, the objective is to identify such regions and spatial patterns, without investigating the factors behind them.

Method

Spatialization of the kdr index values

kdr index values were localized at the geographic coordinates of their respective localities. The geographic coordinates of the Brazilian cities were produced by the Brazilian institute of geography and statistics (Instituto Brasileiro de Geografia e Estatística, IBGE) and have been made available by the Cartography laboratory of the Santa Maria Federal University (UFSM) (http://coral.ufsm.br/cartografia/index.php?option=com_content&view=article&id=20&Itemid=28%20Accessed).

Moran's Eigenvector Maps

Here after, a "significant spatial pattern" is defined as a spatial distribution of values associated with a statistically significant Moran's spatial auto-correlation index (Moran's *I*) value.

To discover significant spatial patterns in the *kdr index* data, the Principal Coordinates analysis of Neighbors Matrices and the Moran's Eigenvector Maps were used – please see Dray et al., 2006 for theoretical considerations and Dray, 2020 for a practical examples using the `adespatial` R package (Dray et al., 2020; R Core Team, 2019).

The method can be summarized as follows:

- defining a weighted neighborhood graph between the observation sites (cities). Such graph represent the "links" between cities, in a broad sense referring to geographical proximity, connectivity, similarities in environmental and/or socio-demographic conditions, etc., and which may explain why cities may share similar statuses of resistance. A graph is represented by a weighted neighborhood matrix;
- decomposing the weighted neighborhood matrix in eigenvectors presenting the following properties: the first eigenvector (the one associated to the greatest eigenvalue) is the one maximizing the Moran'*I* on the given neighborhood graph, the second eigenvector (associated to the next greatest eigenvalue) is the one that also maximizes the Moran'*I* on the given neighborhood graph, with the constraint of orthogonality with the first eigenvector, and so on. Eventually, a set of independent eigenvectors is constructed, ranked in decreasing order of Moran'*I* and from large to local scale auto-correlation;
- Ranking the eigenvectors as a function of the explained variance of the observed *kdr index* values, in the framework of simple linear regressions;
- Selecting the most significant eigenvectors for interpretation.

Weighted proximity graph

In the absence of the *a priori* knowledge of the neighborhood graph best able to explain the *kdr index* spatial distribution, several neighborhood graph and weighting functions were tested. Tested neighborhood graphs were: Delaunay triangulation graph, Gabriel graph, relative neighborhood graph, minimum spanning tree, distance based graphs considering that two cities are connected if their distance is less than a given value *D* (Dray et al., 2006).

Different values of *D* were tested: 1000km, 2000km, 3000km and 4000km. Eight weighted functions were considered: $f_1 = 1-(d/dmax)^{\alpha}$ with $\alpha \in \{1, 2, 5, 10\}$ and $f_2 = 1/d^{\beta}$, with $\beta \in \{0.1, 0.2, 0.5, 1\}$, d being the Euclidean distance between two cities and *dmax* the maximum distance observed on the neighborhood graph.

Consequently, 64 (8 neighborhood graph x 8 weighting functions) were tested, resulting in 64 spatial decompositions (referred to as "models" hereafter) of the spatialized *kdr values*.

Model selection

Dray et al. (2006) propose the corrected Akaike criterion to select the best model. Such a technique provides good prediction results but can select many eigenvectors and does not necessarily highlight the spatial patterns that individually explain the most part of the variance. Here, we focused on few eigenvectors that individually explain large parts of the data variance. Moreover, we did not try to explain the totality of the variance of the *kdr index* values, but the information content associated with significant spatial auto-correlations, at large scales. In practical terms, only spatial eigenvectors associated with positive eigenvalues and that explained at least 15% of the variance were considered. Such eigenvectors are referred to as "patterns" hereafter.

Results and discussion

Fifty five models included patterns that satisfied the above condition: 13 models with 2 patterns and 42 with only 1 pattern. Selected models were ranked according to the percentage of variance of the *kdr index* values explained by the (1 or 2) most explanatory pattern(s) (within the linear regression framework). This was performed by considering separately models with 1 and 2 patterns to make sure not to exclude patterns that, individually, could explain most of the data. The Moran's *I* values of the residuals of the linear regressions were tested to check if the models succeeded in accounting for the totality of the information presenting an overall significant spatial dependence. Eventually, a visual analysis of the spatial patterns exhibited by the top-ranked models permitted to select 5 models that highlight different spatial patterns in the data.

Table TS1 summarizes the main characteristics of the selected models and their components (patterns) and Figure S1 shows the associated proximity graphs and weighting functions. The model 5 appears particularly interesting as it is composed of two components that explain a significant part of the *kdr index* variance (29.6% and 22.0% for the components individually, and 51.6% cumulatively), and because linear regression residuals do not present spatial auto-correlation, demonstrating that the model brings out the major part of the *kdr index* information content that globally present a dependence to space.

Map representations of the spatial patterns are presented Figure S2.

Conclusion

All model components represent well the different patterns that can be intuitively identified by considering the spatial distribution of the *kdr index* values (Figure 4 of the article). In that sense, the analysis succeeds in confirming such intuitions and make the identification of spatial patterns much more objective. Model 5 present particularly interesting statistical characteristics and was chosen as the "best" one. However, such final choice remains quite subjective and the other models should be considered when investigating the resistance determinants, especially since other patterns individually explain more variance than those of the model 5. Such investigation of resistance determinants could be done by studying the relationships between these patterns and the spatial

distribution of environmental (including climatic), socio-demographical, etc. parameters (Roux et al., 2011a, 2011b).

Table TS1: Summary of the characteristics of the selected models and spatial patterns. Model represented in bold is the one that appears in the article.

Model	Graph (see Figure 1)	Weighting function, parameter value (see Figure S1)	Spatial pattern	Percentage of explained variance (%) ($R2x100$)	Moran's I	Moran's I of residuals (linear regression framework)
**1**	Distance based, *D*=1000 km	f_1, α=10	C11	35.3	1.10**(a)	0.12**
**2**	Relative neighborhood	f_1, α=1 (linear)	C21	34.5	1.22**	0.24 (*p*-value=0.001)
**3**	Distance based, *D*=1000 km	f_1, α=2	C31	35.0	1.21**	0.15**
			C32	17.5	0.44**	0.54**
			C31+C32	52.5	0.95**	0.04 (*p*-value=0.015)
**4**	Distance based, *D*=2000 km	f_1, α=1	C41	33.7	0.69**	0.06**
			C42	18.1	0.22**	0.29**
			C41+C42	51.8	0.53**	0.004 (*p*-value=0.141)
**5**	**Distance based, *D*=3000 km**	**f_1, α=1**	C51	29.6	0.37**	0.008 (*p*-value=0.003)
			C52	22.0	0.06**	0.13**
			**C51+C52**	**51.6**	**0.24****	**-0.001 (p-value=0.831)**
(a) **: very significant spatial auto-correlation, *p*-value<<0.001						

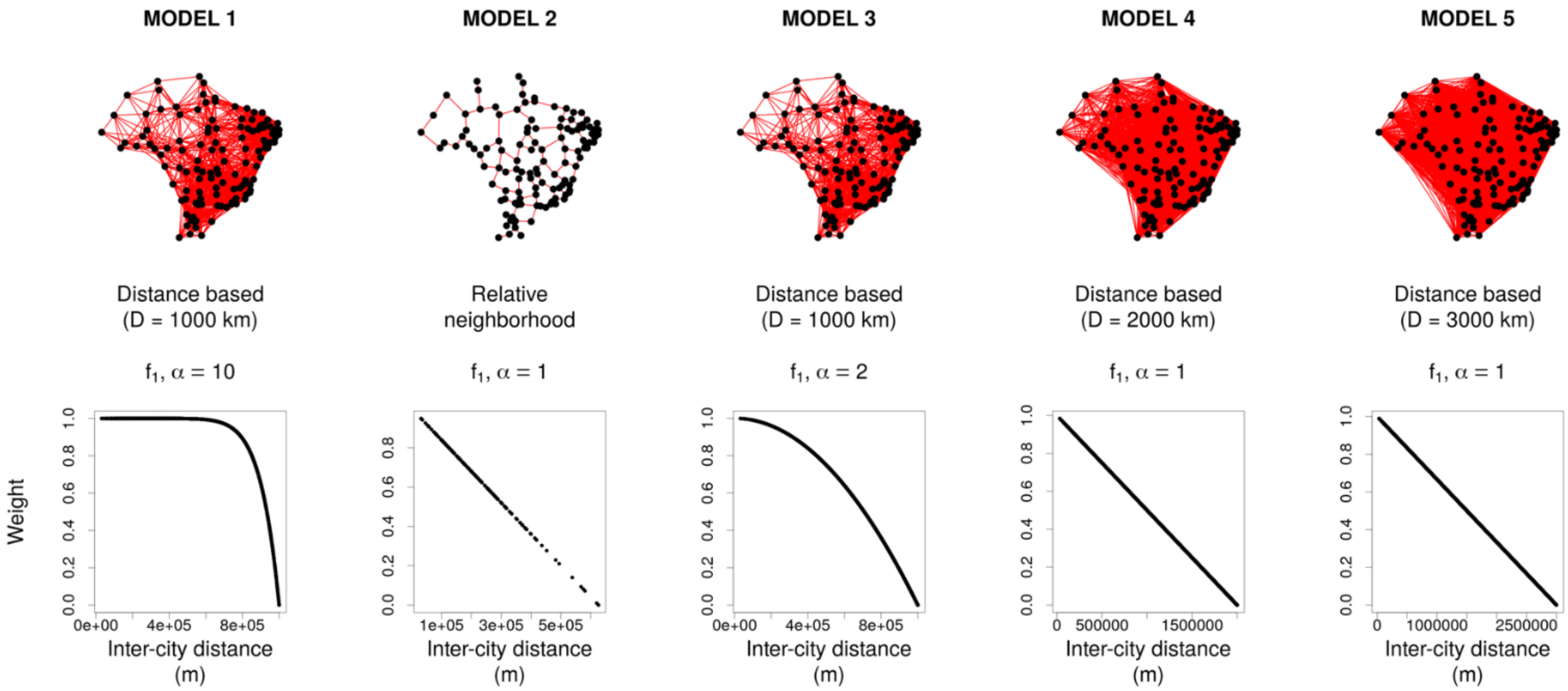

Figure TS1: Neighborhood graphs and weighting functions associated with the selected models

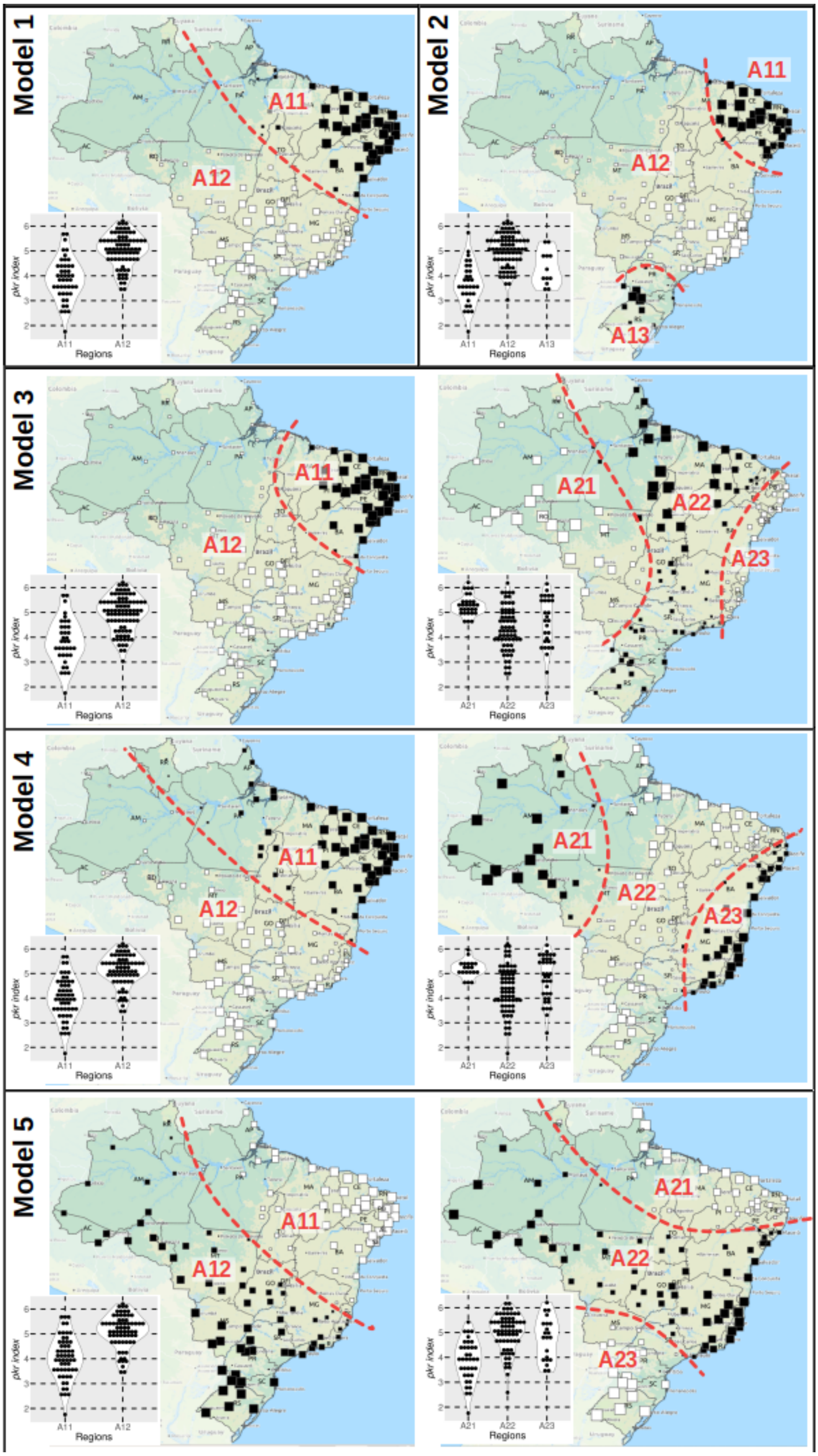

Figure TS2: Map representation of the selected models and components. White and black squares are for negative and positive values of eigenvector components, respectively, and square size is proportional to absolute value of the vector components. The red dashed lines separate regions presenting the same eigenvector value sign and therefore delimited different regions.
The distributions of *kdr index* values are shown as a function of the regions identified.

The background map is the “OSM TF Landscape” product (Maps Thunderforest, Data OpenStreetMap contributors, under license CC-BY-SA 2.0). Maps were generated with the free and open source software (GNU General Public License) QGIS version 3.12.3 (developd by the Open Source Geospatial Foundation Project, http://qgis.org).

References:

Dray S, Legendre P, Peres-Neto PR. Spatial modelling: a comprehensive framework for principal coordinate analysis of neighbour matrices (PCNM). Ecol Modell 2006;196:483–93.

Dray S. Moran's Eigenvector Maps and related methods for the spatial multiscale analysis of ecological data. Tutorial of adespatial R package 2020. https://cran.r-project.org/web/packages/adespatial/vignettes/tutorial.html

R Core Team. R: A language and environment for statistical computing. R Foundation for Statistical Computing, Vienna, Austria 2019. https://www.R-project.org/

Roux E, Venâncio ADF, Girres J-F, Romaña CA. Spatial patterns and eco-epidemiological systems–part I: multi-scale spatial modelling of the occurrence of Chagas disease insect vectors. Geospatial Health. 2011;6:41–51. DOI:10.4081/gh.2011.156

Roux E, Venâncio ADF, Girres J-F, Romaña CA. Spatial patterns and eco-epidemiological systems–part II: characterising spatial patterns of the occurrence of the insect vectors of Chagas disease based on remote sensing and field data. Geospatial health 2011b;6:53–64. DOI:10.4081/gh.2011.157
